# Incomplete binaural restoration in single-sided deafness treated with cochlear implants: insights from compensatory head-orienting behaviors

**DOI:** 10.3389/fnins.2026.1918574

**Published:** 2026-08-12

**Authors:** Hillary A. Snapp, Sandra Prentiss, Sebastian A. Ausili

**Affiliations:** Department of Otolaryngology, University of Miami, Miami, FL, United States

**Keywords:** cochlear implant, orienting behaviors, single-sided deafness, sound localization, spatial hearing

## Abstract

**Introduction:**

Single-sided deafness (SSD) disrupts binaural spatial hearing and may require active compensatory strategies, even for those with cochlear implants (CIs). This study examined spatial hearing adaptations in individuals with single-sided deafness (SSD), including those using cochlear implants (CIs) and unaided SSD, compared to normal-hearing (NH) controls.

**Methods:**

Using a dual-task paradigm, participants performed both simple localization and complex multi-talker speech identification tasks, with continuous tracking of head-orientation dynamics to quantify re-orientations, response promptness, and cumulative movement.

**Results:**

The use of CIs yielded measurable improvements in basic localization tasks; however, these benefits diminished in ecologically valid, dynamic environments. Speech intelligibility remained asymmetric, favoring the better-hearing ear, and performance for CI-side targets collapsed despite favorable signal-to-noise ratios. Listeners with SSD-CI demonstrated rapid, iterative scanning, prolonged orientation sequences, and increased cumulative head movement, suggesting reliance on active compensatory strategies rather than complete restoration of spatial hearing.

**Discussion:**

Current CI technology improves access to sound and spatial hearing for SSD listeners, but it does not fully restore functional binaural hearing in dynamic listening environments. Persistent compensatory head-orienting behaviors indicate reliance on better-ear listening and increased physical effort to position the head in ways that exploit acoustic head-shadow effects, allowing speech access by the better-hearing ear. These findings support the use of ecologically valid assessment models and head-orientation metrics to guide auditory rehabilitation and future innovations for asymmetric hearing loss.

## 1 Introduction

Spatial hearing is fundamental to adaptive human behavior in complex acoustic environments. It relies on the neural integration of interaural time differences (ITDs), interaural level differences (ILDs), and monaural spectral cues to enable sound localization, stream segregation, rapid attentional orienting, and effective communication in dynamic settings (Grothe et al., 2010; van der Heijden et al., 2019; Francl and McDermott, 2022). In dynamic acoustic scenes, where the listener and/or the sound source is in motion, spatial hearing allows for efficient re-orientation and supports auditory scene analysis (van der Heijden et al., 2019; Gohari et al., 2022). These processes demand continuous recalibration of fluctuating cues and motor responses (e.g., head-orienting) to maintain scene awareness and speech intelligibility (Pastore et al., 2020; Hofman et al., 1998).

Hearing loss disrupts spatial hearing abilities, which can lead to significant difficulties in managing the acoustic space. Listeners may lose the ability to locate auditory objects, segregate speech streams, and monitor or follow sound sources. This can ultimately impact listener behavior and lead to increased cognitive load, listening effort, and auditory fatigue, ultimately reducing overall quality of life.

In cases of single-sided deafness (SSD), cochlear implants (CIs) are increasingly used as a form of hearing restoration. While the goal of using CIs for SSD is to restore binaural sensitivity, evidence increasingly suggests that the degraded signal delivered by the implant may limit its effective integration with the high-fidelity input from the intact ear, favoring continued cortical dominance by the normal hearing (NH) ear (Legris et al., 2018; Legris et al., 2020; Lee et al., 2020). This pattern resembles the competitive suppression seen in cross-modal processing (Yu et al., 2019; Chen and Zhou, 2013) and is consistent with reports of incomplete binaural fusion in SSD-CI listeners (Legris et al., 2018; Legris et al., 2020; Lee et al., 2020; de Villars et al., 2025; Muegge and McMurray, 2025; Francart et al., 2018), suggesting that the benefits observed in simple laboratory settings may overestimate real-world spatial hearing restoration.

Spatial hearing cues are frequency-dependent and heavily influenced by signal levels (Bronkhorst and Plomp, 1988; Dirks et al., 2019; Dorman et al., 2015; Arras et al., 2022), making binaural processing particularly vulnerable to profound asymmetry. In SSD-CI listeners, poor ITD transmission, compressed ILDs, and interaural place/timing mismatches limit the benefits of binaural hearing (Dorman et al., 2015; Bernstein et al., 2021; Dorman et al., 2015; Zirn et al., 2015). Localization improves over unaided SSD, but remains well below NH performance (Dirks et al., 2019; Arndt et al., 2011; Dorman et al., 2016). However, in daily life, precise localization may not be essential; right–left discrimination and active head movements often suffice to exploit head-shadow effects and obtain better-ear glimpses for spatial release from masking (Pastore et al., 2020; Hofman et al., 1998; Williges et al., 2019; Rana and Buchholz, 2018; Brungart and Iyer, 2012; Grange and Culling, 2016; Grange et al., 2018). This head-shadow exploitation represents a key behavioral adaptation in asymmetric hearing, increasing listening effort but enabling functional speech perception (Grange and Culling, 2016; Grange et al., 2018; Su et al., 2025; Snapp and Ausili, 2020).

Despite some CI benefit for speech intelligibility with concurrent talkers (Bernstein et al., 2022; Bernstein et al., 2022), the role of the implant in dynamic, multi-talker environments, where cues fluctuate rapidly and tax neural integration and demand compensatory motor strategies, remains poorly understood. Current spatial hearing assessments dramatically underestimate these real-world challenges. In dynamic scenes, remnant asymmetries likely produce erroneous or unstable binaural cues, reinforcing better-ear listening and impairing rapid stream segregation and attentional orienting. It has been suggested that CIs provide greater confidence in decision-making regarding target location (Bernstein et al., 2022; Valzolgher et al., 2020). For instance, in dynamic spatial listening, rapid identification of auditory targets may allow listeners to more efficiently re-orient to facilitate speech perception in the presence of competing speech streams. Evidence supports that head movements improve localization abilities (Grange and Culling, 2016; Noble, 1981; Thurlow and Runge, 1967; Gessa et al., 2025). However, compensatory mechanisms, specifically head movements, performed by hearing-impaired listeners remain unclear.

To address this gap, the present study used a dual-task paradigm combining dynamic localization and speech intelligibility in moving multi-talker scenes. We aimed to (1) quantify spatial hearing deficits in SSD-CI listeners under dynamic conditions compared to untreated SSD listeners (SSDa) and NH controls, and (2) characterize their behavioral compensatory strategies (e.g., head movements). We hypothesized that SSD-CI participants rely on better-ear listening and increased use of compensatory head-orienting behavior.

## 2 Methods

### 2.1 Participants

A total of 16 listeners with acquired SSD (SSDa) (4 men and 12 women; Mean age = 48.03 and SD = 15.2 years) and 9 listeners with acquired SSD who had CIs (5 men and 4 women; Mean age = 52.8 and SD = 14.1 years) participated in the study. The duration of deafness was a median of 3.8 years (IQR, 0.8–11.20; range, 0.2–56). The CI group was tested with CI on (SSD-CI) and with CI off (SSD-CIoff). All listeners had audiometric thresholds (<20 dB HL) from 125 Hz to 6 kHz in the better hearing ear, while experiencing severe to profound hearing loss in the impaired ear (i.e., the SSD ear). All CI-implanted SSD participants had at least 6 months of CI listening experience, with a median of 1.6 years (IQR, 1–2.6, range, 0.7–3.4). A control group of bilaterally NH listeners (seven men and nine women; Mean age = 29.7 and SD = 7.6 years) was also included. All participants were naïve to the experiments. All experimental procedures were carried out in accordance with relevant guidelines and regulations for research involving human participants. The study protocol was reviewed and approved by the Institutional Review Board of the University of Miami (IRB Protocol No. 20211149). Written informed consent was obtained from all participants prior to enrollment in the study.

### 2.2 Experimental set-up

The experiments took place in a sound-attenuated auditory booth (4.3 × 4.3 × 2 m) with a spherical array of 72 speakers (e301, KEF, Maidstone, England) of 1.3 m in radius. The audio signals were driven by a Focusrite RedNet MP8R (Focusrite, High Wycombe, England) connected to Sonible d:24 multi-channel amplifiers (Sonible, Graz, Austria) with a sampling rate of 44.1 kHz. A camera system with a total of 6 trackers (Flex 3 Optitrack, Natural Point, Corvallis, USA), together with a custom headband with reflective markers, was used to obtain head orientation measurements. The head-tracking system was calibrated prior to the start of the experiment for each participant. Data quality was examined following calibration by having participants complete target validation.

Stimuli presentation was implemented in MATLAB (ver. R2023a, The MathWorks, Natick, USA) using the Psychtoolbox extension (Brainard, 1997; Pelli, 1997; Kleiner et al., 2007). Lab Streaming Layer library^1^ was implemented for device time synchronization.

#### 2.2.1 Stimuli

Sound localization stimuli consisted of 150 ms broadband Gaussian white noise bursts (BB, 0.2–20 kHz) presented at 50 dBA, 60 dBA, and 70 dBA, totaling 138 trials presented randomly from 46 speakers spanning ±90° in azimuth and ±30° in elevation.

Spatial speech perception stimuli consist of target sentences from the coordinated response measure (CRM) corpus (Bolia et al., 2000). All CRM sentences have the same structure: “ready (call-sign) go to (color) (number) now,” and consist of eight possible call signs (Arrow, Baron, Charlie, Eagle, Hopper, Laker, Ringo, Tiger), four colors (blue, red, green, white), and eight numbers (Grothe et al., 2010; van der Heijden et al., 2019; Francl and McDermott, 2022; Gohari et al., 2022; Pastore et al., 2020; Hofman et al., 1998; Legris et al., 2018; Legris et al., 2020). The CRM stimuli are broadband in nature and embedded in multiple competing speech streams, allowing for the investigation of how listeners may leverage available spatial perception cues in complex environments while managing more than one task simultaneously (i.e., target identification, target localization, and speech intelligibility).

#### 2.2.2 Test paradigm

**Figure 1:**
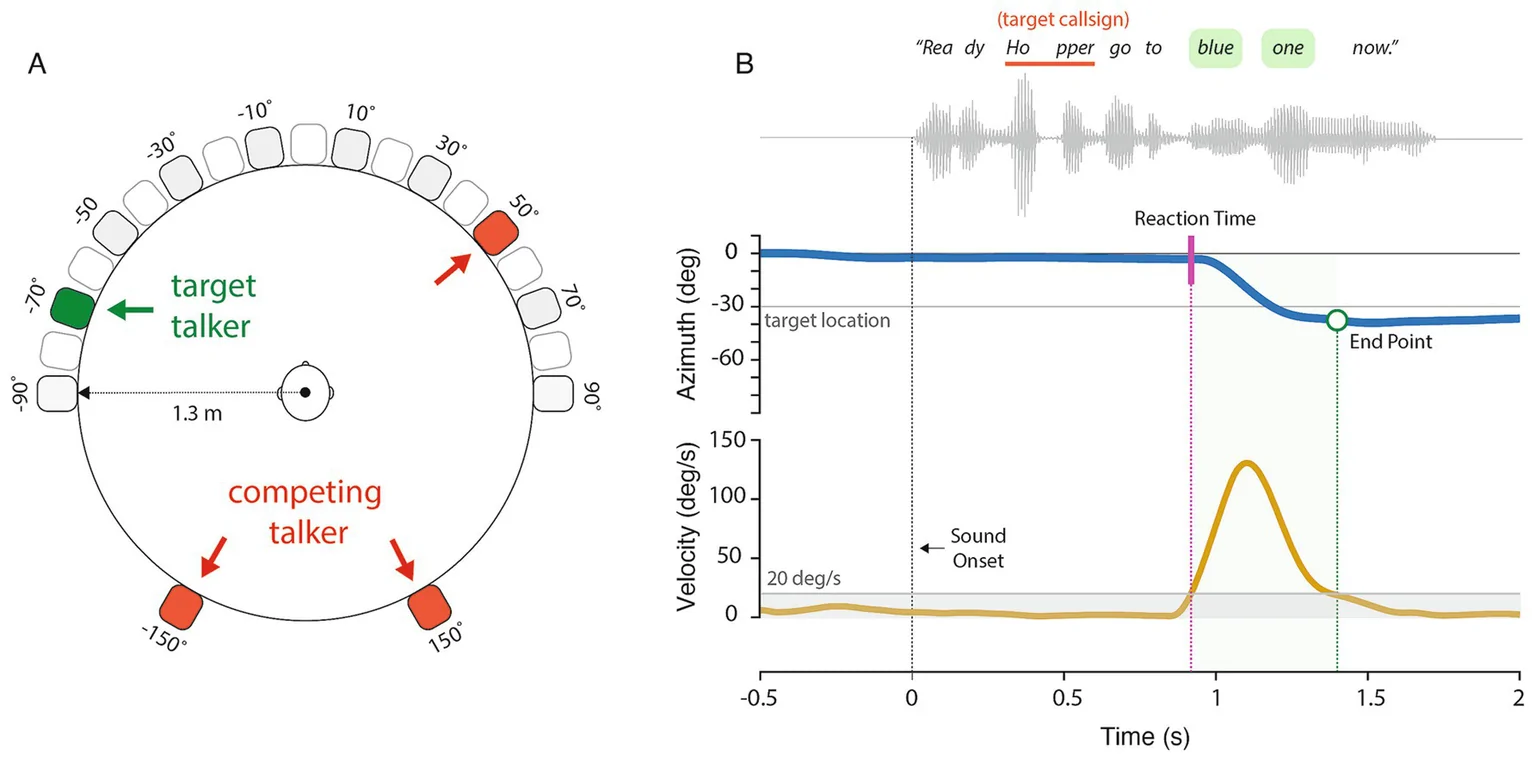
**(A)** Sample target-to-masker configuration for the dynamic spatial speech perception task; target speakers are indicated in grey; **(B)** sample target stimulus from the CRM corpus is presented relative to the head-oriented response. Upper panel shows head position in azimuth, and the lower shows the velocity of head movement. Reaction time is calculated as the time between the stimulus onset and the initiation of the head movement.

Participants were seated in the center of the array in a totally darkened sound-attenuated room to remove response bias and eliminate visual cues during the experiment. Figure 1 presents the testing paradigm for the dynamic spatial speech perception experiment. Participants were instructed to listen for the target talker, who was identified by the call sign “Hopper.” Target stimuli were randomly presented in the front hemifield at signal-to-noise ratios (SNRs) of +3, +5, and +8 dB against three interfering male maskers. Each masker consisted of a full CRM sentence featuring a call sign, color, and number distinct from the target sentence. Maskers were presented at 70 dBA in quadrants alternating with the target talker. Listeners were asked to (1) locate the source of the target talker and (2) report (identify and recall) the associated color/number (e.g., “Blue” “One”).

### 2.3 Data analysis

For SSD participants, azimuth was sign-flipped on an individual basis such that positive values represent the better-hearing (non-implanted) ear side and negative values the impaired (deaf or implanted) ear side to enable data analysis across participants. Although stimuli were presented in azimuth (±90°) and elevation (±30°), the present analysis focuses exclusively on the azimuthal component of the localization response to directly address our primary hypotheses regarding better ear listening strategies, which fundamentally rely on lateralized acoustic cues in the horizontal plane. Linear regression was performed to assess the sound localization stimulus–response relationship in azimuth using the least-squares error criterion on the location data points. The regression was performed on the target angle (*α*_T_) and the response angle ( $\alpha_{R}$ ) as follows:

$$\alpha_{R}=b+g\times \alpha_{T}$$ (1)

Here, g represents the slope or gain of the best-fit regression line (dimensionless), and b represents the intercept or bias (in degrees). Response variability, *σ*, was quantified by calculating the standard deviation of the residuals around the best-fit line.

Mean absolute error (MAE) was calculated across trials to quantify localization response accuracy:

$$\mathrm{MAE}=\frac{1}{N}\sum_{n=1}^{N}|\alpha_{R}^{n}-\alpha_{T}^{n}|$$ (2)

With *N* the total number of trials and n each individual trial.

To measure the promptness of the localization trials, reaction times (RT; in milliseconds) were calculated as the difference in time between the onset of head movement and the onset of the target sound. We then computed the average of the reciprocal of each participant’s reaction time for each stimulus to obtain s-1 values as a measure of promptness (1/RT).

In addition to the stimulus–response relationship, we analyzed the number of head-orienting adjustments (i.e., head position changes in azimuth over time) by the listener from initial reaction to endpoint. A head-orienting response was defined as the point at which the velocity profile (first derivative of head position) exceeded 20 degrees per second. To determine whether head-orienting behavior was associated with performance, Spearman’s rank correlations (chosen given the non-normal distributions of the head-movement metrics) were computed between subject-level head-movement metrics (re-orientations, cumulative orientation, response promptness) and subject-level performance metrics (gain, r^2^, MAE, percent-correct identification), with Benjamini–Hochberg FDR correction for multiple comparisons.

A linear mixed model was conducted to examine the main effects of experimental group (NH, SSD-CI, SSD-CIoff, and SSDa), stimulus presentation side (hemifields with azimuths >20° and <−20° azimuth), and participant’s age on sound localization performance. Dependent variables included gain, r2, bias, MAE, and promptness. In addition, for the dynamic spatial speech perception experiment, the dependent variables were the percent correct color/number identification and the number of head orientations, with SNR included as an independent factor. Participant ID was entered as a random intercept to account for the repeated within-subject measurements contributed by the SSD-CI group under CI-on and CI-off conditions, while NH and SSDa participants contributed independent between-subject observations. All statistical tests were conducted at a significance level of *p* < 0.05. To further examine specific differences, *post hoc* pairwise comparisons were performed based on estimated marginal means, applying Bonferroni-adjusted significance levels to account for multiple testing. All analyses were performed in SPSS Statistics (version 31, IBM Corp., Armonk, NY, USA).

## 3 Results

### 3.1 Horizontal sound localization

**Figure 2:**
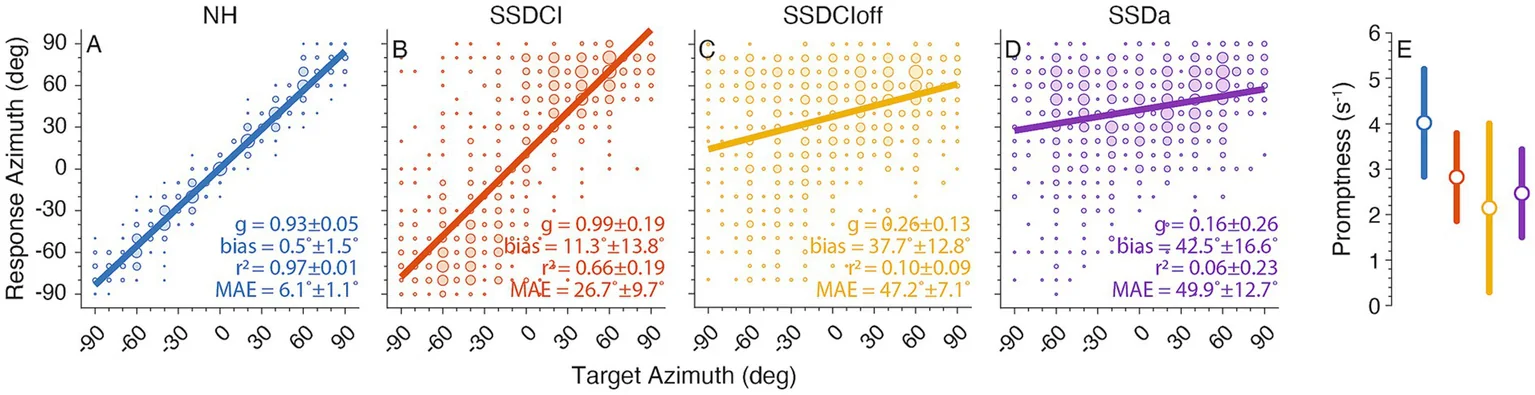
Target response plots present the pooled azimuth localization data of **(A)** NH controls (blue), **(B)** acquired SSD listeners with (red) and **(C)** without CI (yellow), and **(D)** non-implanted acquired SSD (purple). Inset provides gain, bias, the coefficient of determination (*r*^2^), and MAE. **(E)** Mean and standard error of localization promptness are depicted for each group.

Horizontal sound localization performance for broadband noise bursts presented in quiet is summarized in Figure 2. NH controls demonstrated high accuracy (r^2^ = 0.97) and low MAE (6.6°). Linear mixed-effects models revealed robust main effects of group on all measures: azimuth gain (*F*[3,275] = 38.54, *p* < 0.001), bias (*F*[3,275] = 22.92, *p* < 0.001), *r*^2^ (*F*[3,52.33] = 208.55, *p* < 0.001) and MAE (*F*[3,58.34] = 136.93, *p* < 0.001). Age did not significantly predict gain (*F*[1,275] = 0.39, *p* = 0.533), bias (*F*[1,275] = 0.24, *p* = 0.621), *r*^2^ (*F*[1,36.91] = 0.32, *p* = 0.575), or MAE (*F*[1,39.90] = 0.01, *p* = 0.987).

*Post hoc* comparisons showed significant improvements in SSD-CI compared to SSD-CIoff: gain (mean difference = 0.43, SE = 0.096, *p* < 0.001, 95% confidence interval [0.175, 0.686]), bias (mean difference = −14.03, SE = 6.3, *p* = 0.018, 95% confidence interval [−26.47,–1.59]), *r*^2^ (mean difference = 0.122, SE = 0.022, *p* < 0.001, 95% confidence interval [0.062,0.181]), and MAE (mean difference = −23.21, SE = 2.07, *p* < 0.001, 95% confidence interval [−28.73, −17.7]). MAE improved from 47.2° ± 7.1°(CI off) to 26.7° ± 9.7° (CI on). Despite this benefit, SSD-CI performance remained markedly inferior to NH listeners (MAE mean difference = −18.79, SE = 2.73, *p* < 0.001, 95% confidence interval [−26.27, −11.31]; r^2^mean difference = 0.63, SE = 0.035, *p* < 0.001, 95 confidence interval [0.535, 0.731]).

A close review of individual response patterns (Figure 2B) revealed that SSD-CI listeners primarily lateralized rather than localized sounds, with responses clustering between ±50°–90° irrespective of true azimuth, contrasting the linear distribution observed in the NH controls (Figure 2A).

Stimulus level (50, 60, 70 dB SPL) did not affect gain (*F*[2, 275] = 1.42, *p* = 0.244) or *r*^2^ (*F*[2, 245.35] = 3.06, *p* = 0.058) but increased bias (*F*[2, 275] = 7.46, *p* < 0.001), and MAE (*F*[2, 248.98] = 3.68, *p* = 0.027), Significant Group × Side interactions emerged across all metrics (all *p* < 0.002), reflecting asymmetry between the better and impaired ears in the SSD groups. A three-way Group × Side × Level interaction reached significance only for MAE (*F*[6, 242.99] = 3.26, *p* = 0.004), indicating that SSD-CI listeners derived level-dependent improvements selectively on the implanted side at higher intensities. *Post hoc* analyses revealed that while NH listeners maintained low MAE values across all levels and sides (6.1 ± 1.1°), SSD groups exhibited NH side-dependent improvements with level (50 dB: 24.6 ± 7.9°, 60 dB: 26.7 ± 8.5°, 70 dB: 31.2 ± 9.8°).

Response promptness differed significantly between groups (*F*[3,89] = 10.84, *p* < 0.001). NH listeners responded fastest to localization stimuli (Figure 2E), outperforming all SSD groups (*p* < 0.01). No significant differences were found among SSDa, SSD-CI, and SSD-CIoff groups (*p* > 0.5).

### 3.2 Dynamic spatial speech perception

**Figure 3:**
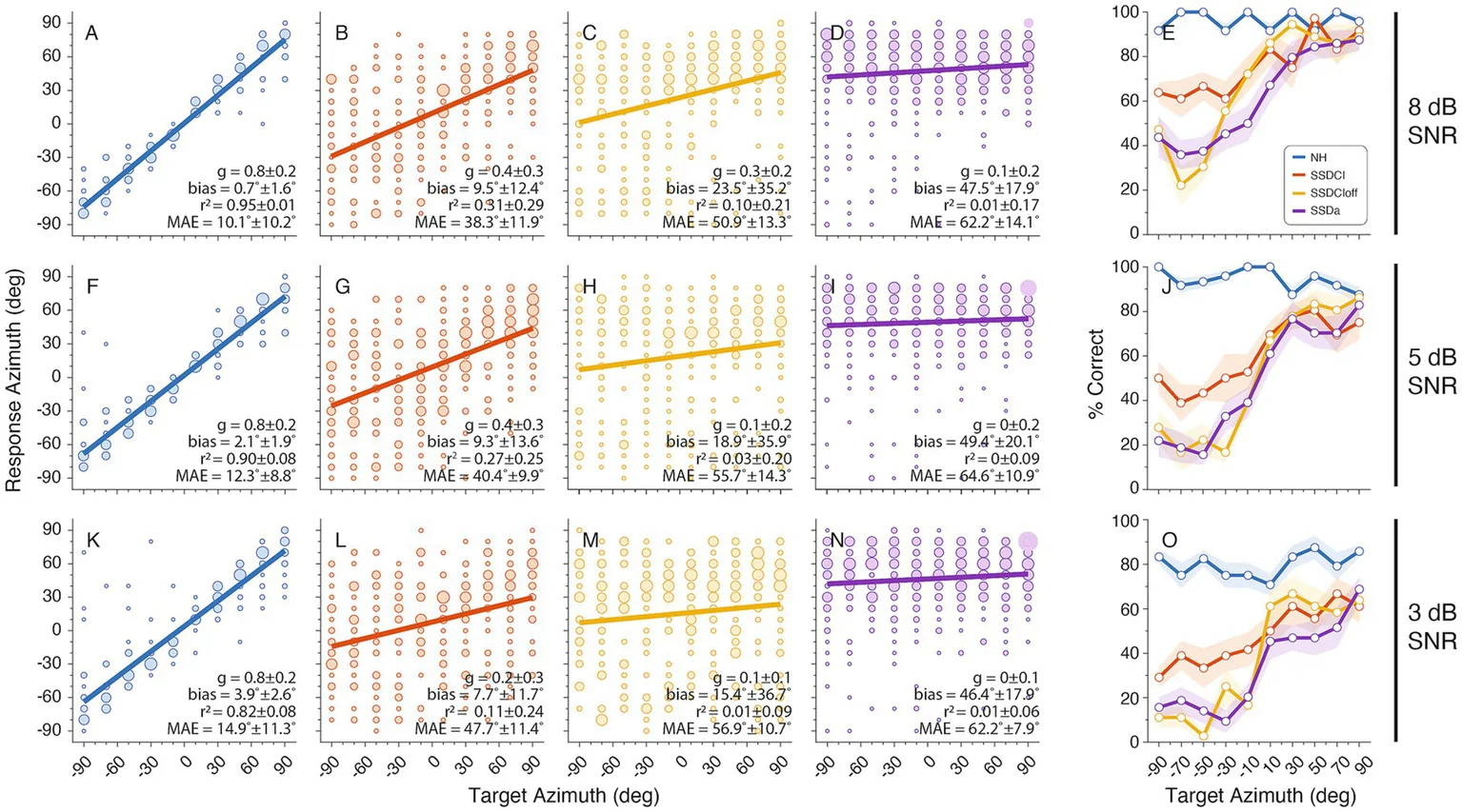
Results of the dynamic spatial speech perception task by SNR (rows). Localization of the target speech stream is shown in columns for: NH (**A,F,K**; blue), SSD-CI (**B,G,L**; red), SSD-CIoff (**C,H,M**; yellow), and SSDa groups (**D,I,N**; purple). The last column presents mean speech intelligibility and its standard error as a function of target location in azimuth for all groups (**E,J,O**).

Localization and speech intelligibility performance during the dual-task paradigm (target-talker segregation + localization + call-sign recall) are presented in Figure 3. Main effects of group were observed for gain (*F*[3,335] = 91.49, *p* < 0.001), bias (*F*[3,38.03] = 13.96, *p* < 0.001), *r*^2^ (*F*[3,39.85] = 150.26, *p* < 0.001) and MAE (*F*[3,37.3] = 43.17, *p* < 0.001). In this task, age did not significantly predict gain (*F*[1,335] = 2.35, *p* = 0.126), bias (*F*[1,27.00] = 0.01, *p* = 0.928), *r*^2^ (*F*[1,27.67] = 1.33, *p* = 0.259), MAE (*F*[1,26.43] = 0.45, *p* = 0.510), percent correct speech identification (*F*[1,26.30] < 0.01, *p* = 0.967), response promptness (*F*[1,27.02] = 0.49, *p* = 0.488), or number of head-orientations (*F*[1,27.05] = 0.54, *p* = 0.468). NH listeners maintained good localization abilities across SNRs (Figures 3A,F,K), whereas localization in the SSD groups was severely degraded (Figures 3B–D,G).

CI use conferred modest benefits over the CI-off condition: gain (mean difference = 0.2, SE = 0.04, *p* < 0.001, 95% confidence interval [0.90, 0.31]), bias (mean difference = −13.18, SE = 3.90, *p* = 0.005, 95% confidence interval [−23.55, −2.81]), *r*^2^ (mean difference = 0.09, SE = 0.02, *p* < 0.001, 95% confidence interval [0.03, 0.14]), and MAE (mean difference = −12.49, SE = 2.46, *p* < 0.001, 95% confidence interval [−19.01, −5.97]) in SSD listeners. However, SSD-CI localization remained poor, with low r^2^ and response patterns indicative of lateralization rather than true localization. Untreated SSDa listeners showed near-zero gain (*M* = 0, SD = 0.1) and extreme bias toward the hearing side (*M* = 47.8°, SD = 18.3°).

Speech intelligibility was strongly influenced by target side (*F*[1,208.08] = 119. 86, *p* < 0.001), with a significant Group × Side interaction, (*F*[3,208.08] = 14.29, *p* < 0.001; Figures 3E,J,O). When the target talker was presented to the better ear, performance was comparable across SSD groups (53–89% correct) and independent of SNR (SNR × Group × Side: *F*[6,297.77] = 0.82, *p* = 0.632). In contrast, intelligibility collapsed when the targets originated from the impaired/CI side, even in the most favorable conditions (+8 dB SNR). The NH listeners maintained ≥90.1% ± 3.2% correct across the entire horizontal plane.

### 3.3 Effect of task complexity: quiet broadband noise vs. target talkers in competing speech

To assess the effect of increased task complexity on spatial acuity, we compared performance between the simple broadband-noise localization task (Quiet, Figure 2) and the complex multitalker localization task requiring target-talker segregation and speech recall (Dynamic Spatial Speech Perception, Figure 3).

Mixed linear model analyses revealed no significant main effect of the experiment on any performance measures. Specifically, gain did not differ between conditions [*F*(1, 15) = 2.479, *p* = 0.136], nor did bias [*F*(1,20) = 0.003, *p* = 0.956], *r*^2^ [*F* (1,20) = 0.170, *p* = 0.684], or MAE [*F*(1, 15) = 4.242, *p* = 0.057]. Thus, NH listeners maintained equivalent localization precision, accuracy, and reliability when task demands increased from simple sound localization to active target segregation in competing speech.

In contrast to NH participants, increasing task complexity produced robust degradation across multiple metrics in SSD-CI. A significant main effect of experiment was observed for gain [*F*(1,26) = 2.479, *p* = 0.020], *r*^2^ [*F*(1,26) = 19.035, *p* < 0.001], and MAE [*F*(1,26) = 34.858, *p* < 0.001], indicating reduced sensitivity, poorer model fit, and larger absolute errors in the spatial speech perception condition. Bias showed no main effect of experiment [*F*(1,26) = 2.556, *p* = 0.122].

Significant trend-level experiment × side interactions further revealed lateralized vulnerability. For gain, the interaction was significant [*F*(1,26) = 6.775, *p* = 0.015], driven by greater degradation on the CI side. A similar pattern emerged for MAE [*F*(1,26) = 6.332, *p* = 0.057] and marginally for bias [*F*(1,26) = 3.391, *p* = 0.077]. A *post-hoc* inspection confirmed that performance on the CI side was significantly lower than on the better-hearing side in the complex task, although differences were less or absent in the simple sound localization condition (Figure 2).

### 3.4 Head-orientation strategies in spatial speech perception

**Figure 4:**
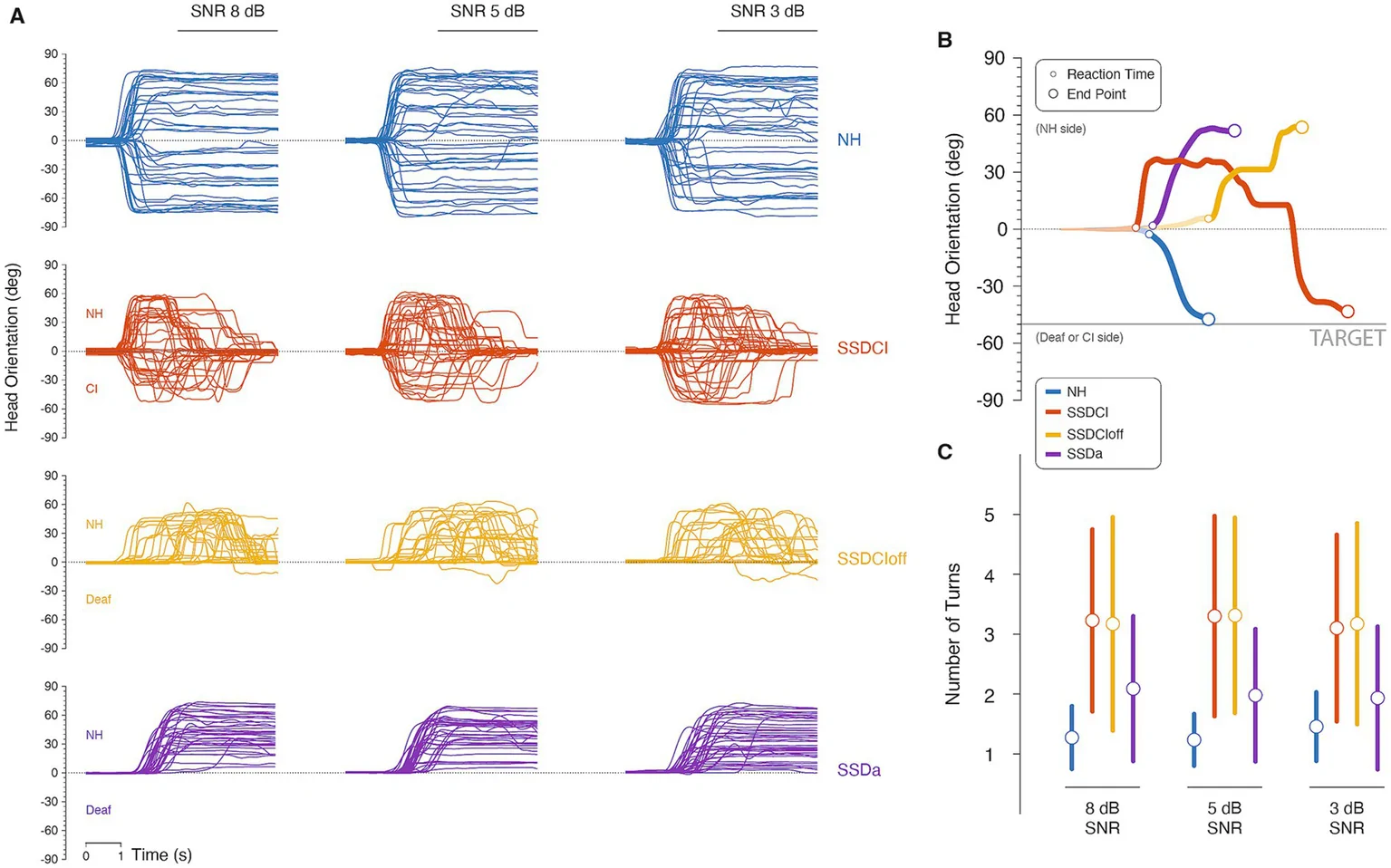
Head movement dynamics from the spatial speech perception task. **(A)** Individual representations of head traces over time for the CRM corpus task are shown for NH (blue), SSD with CI on (red) and CI off (yellow), and SSD-acquired groups. **(B)** For simplicity and direct comparison, single-trial head trace examples are also depicted. **(C)** Last, the mean and standard deviation of the number of turns produced are shown by each group and SNR.

To explore the behavioral strategies listeners use to facilitate spatial speech perception, we analyzed the head-orientation patterns across the groups (Figure 4). All experimental groups (SSDa, SSD-CI, SSD-CIoff) performed significantly worse than NH listeners across all head-movement metrics: number of re-orientations (compensatory head turns), response promptness (reaction times), and cumulative orientation (total spatial coverage in degrees). Specifically impaired listeners took substantially longer to reach the perceived target location, scanned significantly more total degrees, required more compensatory re-orientations, and showed significantly larger localization errors than NH controls. CI use did not improve performance on any metric.

Head-movement trajectories revealed distinct compensatory strategies. NH listeners executed rapid, single-step orientations to the target (Figure 4A). As illustrated in Figure 4C, listeners with SSD required significantly more head re-orientations (*M* = 2.6 ± 0.7) than NH controls (*M* = 1.6 ± 0.4), as indicated by a main effect of group (*F*(3,38.07) = 3.75, *p* = 0.019). Age did not significantly predict the number of head re-orientations (*F*[1,27.05] = 0.54, *p* = 0.468). Notably, unaided SSD participants demonstrated a pronounced bias toward the better ear (see example Figure 4A; yellow). When CI-aided (Figure 4A; red), localization behavior changed, and the SSD listener made use of the entire horizontal plane, but performance remained poor. SSD-CI re-orientations (*M* = 2.6, SD = 0.3) were not significantly different from the CI-off condition (mean difference = 0.028, SE = 0.048, *p* = 1.00, 95% confidence interval [−0.154,0.098]). SSDa listeners (purple tracings), although inaccurate, executed rapid single-step orientations to the target similar to NH, with fewer re-orientations than SSD-CI recipients (SSDa: *M* = 2.0, SD = 0.5; mean difference from NH = -0.338, SE = 0.286, *p* = 1.00, 95% confidence interval [−1.149,0.473]).

**Figure 5:**
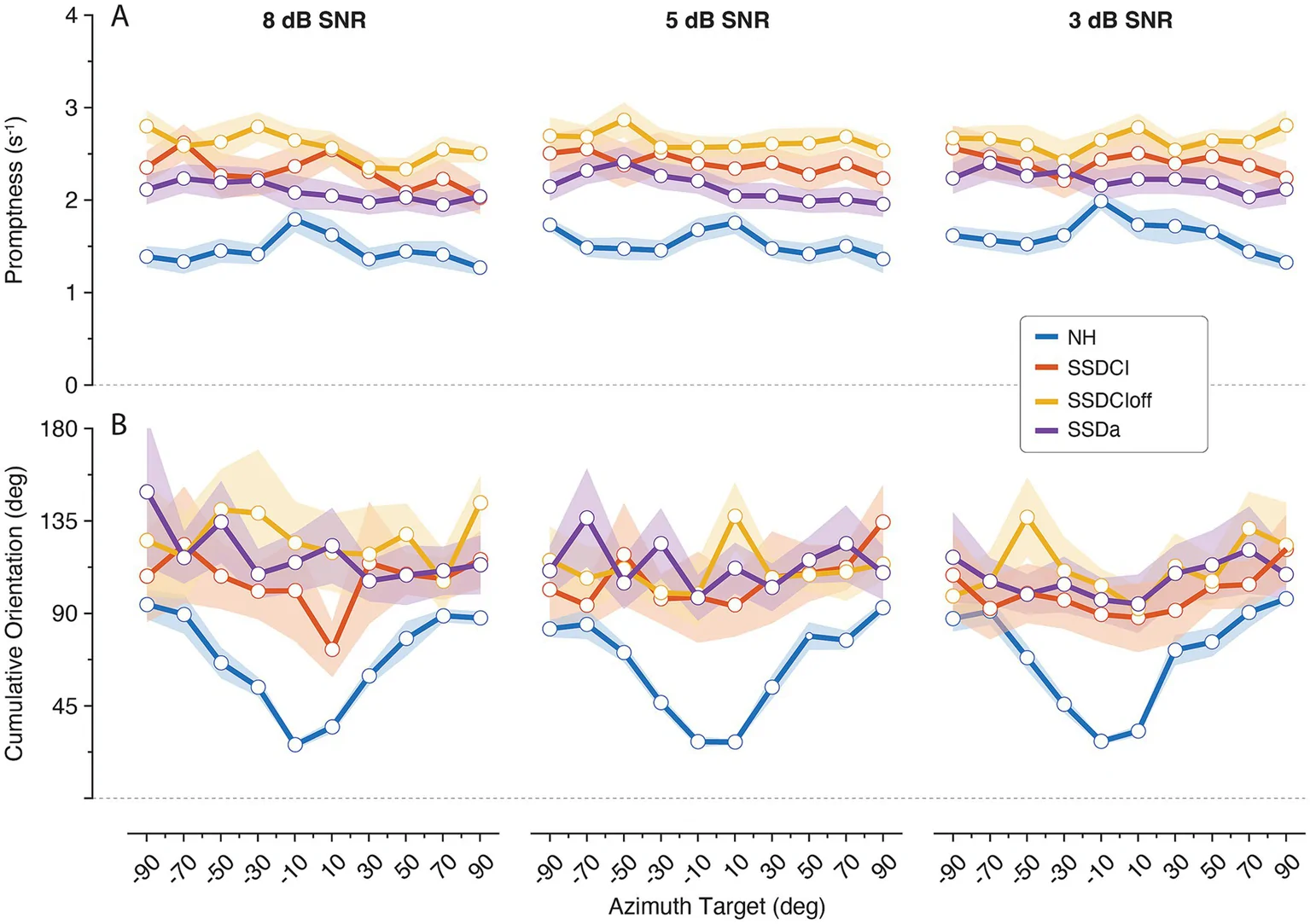
**(A)** Mean response promptness (inverse of reaction time) and cumulative orientation **(B)** across subjects for each SNR and listening group are presented on the horizontal plane. Shaded areas show the standard error of the mean.

Response promptness showed a reversal pattern compared to localization in quiet (Figure 5A). SSD listeners initiated head movements significantly faster than NH controls [main effect: *F*(3,38.04) = 22.49, *p* < 0.001], with SSDa and SSD-CIoff being the fastest (2.5 s^−1^ and 2.17 s^−1^, respectively). Similar to previous outcomes, age did not significantly predict response promptness [*F*[1,27.02] = 0.49, *p* = 0.488]. Furthermore, promptness was unaffected by SNR [*F*(2,284.07) = 0.627, *p* = 0.811] or target side [*F*(1,91) = 1.27, *p* = 0.321], suggesting that rapid, inaccurate orienting reflects a low-confidence search strategy rather than efficient localization.

As expected, cumulative orientation (total head movement in degrees from response onset to stop) in NH reflects expected binaural function: signals presented directly in front elicit minimal head movement, whereas signals positioned off-center prompt head movements that closely match the target azimuth in degrees (Figure 5B). None of the SSD groups exhibited the expected pattern of head movement observed in NH listeners; instead, their cumulative orientation was significantly greater [*F*(3,38.02) = 15.44, *p* < 0.001], indicating reliance on compensatory search strategies. Age did not significantly predict cumulative orientation [*F*[1,27.00] = 0.04, *p* = 0.851]. CI use did not reduce the total head movement leveraged by SSD listeners to locate the perceived target (*p* > 0.721). No effect was observed across SNR [*F*(2,82.23) = 1.972, *p* = 0.237] or group-SNR interaction [*F*(6,82.23) = 0.682, *p* = 0.701]. Overall, greater cumulative orientation was associated with poorer speech intelligibility (*ρ* = −0.46, p_FDR = 0.027) and lower localization r^2^ (ρ = −0.44, p_FDR = 0.027). However, these associations were substantially attenuated and no longer significant once group membership was accounted for (ρ = −0.25, *p* = 0.14 for speech intelligibility; ρ = −0.09, *p* = 0.62 for *r*^2^). Within groups, cumulative orientation was not significantly associated with either measure in SSD-CI (speech intelligibility ρ = 0.17, *p* = 0.67; *r*^2^ ρ = 0.55, *p* = 0.13) or SSD-CIoff listeners (speech intelligibility ρ = −0.07, *p* = 0.86; *r*^2^ ρ = −0.20, *p* = 0.61). Only unaided SSDa listeners showed a significant negative association, and only with speech intelligibility (ρ = −0.56, *p* = 0.024; *r*^2^ ρ = −0.41, *p* = 0.12). No other head-movement metric (re-orientations, promptness) was significantly associated with performance.

## 4 Discussion

In agreement with prior reports (Dirks et al., 2019; Arndt et al., 2011; Dorman et al., 2016), SSD-CI listeners demonstrate significant improvement in perceiving and lateralizing brief noise bursts in the horizontal plane. However, this improvement is primarily attributed to enhanced right–left discrimination rather than precise azimuthal localization. Response patterns reveal that SSD-CI listeners consistently assign stimulus position into broad 50–90° hemifield clusters, underscoring that cochlear implantation supports coarse hemifield sensitivity rather than fine-grained binaural spatial acuity (Dirks et al., 2019).

Simple localization of brief, isolated stimuli in quiet markedly under-represents the challenges of everyday acoustic environments. Speech streams are long in duration and complex in their spectrotemporal structure. Moreover, speech streams are frequently embedded within competing maskers whose relative intensities and spatial positions fluctuate dynamically, thereby imposing substantial demands on the rapid binaural comparison and auditory streaming processes. Our ecologically valid dual-task paradigm required participants to locate sounds and recognize speech simultaneously amid noise. The CI benefit observed under simple conditions (Figure 2) was lost as task demands escalated, resulting in substantially reduced performance in more challenging environments (Figure 3). Even at the most favorable +8 dB SNR, SSD-CI localization accuracy was severely degraded and scattered (*r*^2^ ≤ 0.31) with pronounced better-ear dominance. Speech intelligibility was largely preserved only for targets on the NH side (90.1% ± 3.2% intelligibility). In contrast, intelligibility collapsed for targets presented on the CI side. CI activation yielded only marginal intelligibility gains on the impaired side without improving spatial accuracy, revealing a strategic behavioral shift suggesting listeners may favor monaural better-ear capture rather than relying on binaural integration. This is consistent with a behavioral adaptation to unreliable interaural cues that prioritizes speech comprehension over spatial accuracy in complex acoustic scenes.

Head-movement patterns provide behavioral evidence consistent with incomplete binaural restoration under dynamic listening conditions. At 0° azimuth, where ITD and ILD cues are nominally zero, NH participants exhibited virtually no head movement (Figure 5B), supporting immediate central registration of a fused, stable midline perception. For off-center targets, NH listeners showed target-dependent cumulative orientation that closely approximated target azimuth (Figure 5B). Conversely, all SSD conditions (unaided, CI-on, CI-off) showed persistent, expansive scanning (Figure 4B) even at true midline (Figure 5B), suggesting active searching to manage spatial uncertainty. The absence of age, SNR, and CI-on versus CI-off effects suggests that increased cumulative orientation was not simply attributable to age, acoustic difficulty, or immediate CI activation, but instead characterized SSD listening behavior more broadly. This searching strategy in SSD-CI may reflect reliance on monaural head-shadow (Grange and Culling, 2016; Grange et al., 2018; Su et al., 2025; Snapp and Ausili, 2020) to aid localization and stream extraction, rather than the effective use of binaural cues. The observed turn-pause-turn-pause trajectories (Figure 4) are consistent with active sampling of the acoustic scene. Listeners orient to maximize signal capture in the intact ear, pause for cue re-analysis, then reorient as needed. Although SSD-CI listeners were more successful than unaided listerners in locating targets, they employed significantly more head movements (corrections) to resolve speech streams. These findings align with prior work demonstrating that impaired listeners use reversals and multiple corrections to improve accuracy (Brimijoin et al., 2010), leveraging head orientation to exploit the head-shadow effect and optimize SNR at the better ear (Grange and Culling, 2016; Grange et al., 2018; Su et al., 2025; Snapp and Ausili, 2020; Gessa et al., 2025).

The elevated cumulative orientation at 0° azimuth (Figure 5B) serves as a distinguishing behavioral marker. Minimal exploration would be expected in a functional binaural system when interaural cues signal midline, consistent with the pattern observed in our NH listeners. Persistent scanning instead suggests unresolved spatial ambiguity and continued reliance on the intact ear as the primary perceptual reference. These iterative movements represent a significant behavioral adaptation, shifting listening from a passive activity into an active, physically demanding process.

In asymmetric hearing, unreliable or erroneous cues from dynamically changing sound sources may disorient listeners, increasing the need for active repositioning to resolve ambiguity. In SSDa listeners, although inaccurate, they appeared to execute fewer, larger, and more direct orientations to expose the better-hearing ear, consistent with the strong better-ear bias observed in localization responses (Figure 4A). Thus, similar cumulative orientation across SSD groups may reflect different orienting patterns: one larger oriented response in SSDa versus multiple smaller adjustments in SSD-CI listeners. However, neither pattern reliably supported improved speech intelligibility. Rather, in SSDa listeners, greater cumulative orientation was associated with poorer speech understanding, suggesting that larger head movements may reflect greater listening difficulty rather than a successful orienting strategy. Although CI use improved speech perception in SSD-CI listeners, this benefit was not explained by the observed head-orientation behaviors.

Response initiation rates revealed additional group-level differences (Figure 5). NH listeners were slower (less prompt; 1.49 ± 0.38 s^−1^) than SSD-CI aided (2.17 ± 0.23 s^−1^, *p* = 0.023) or unaided (2.5 ± 0.19 s^−1^, *p* = 0.002), and SSDa (2.1 ± 0.52 s^−1^, *p* = 0.002). This pattern suggests that NH listeners may employ a stepwise strategy whereby they listen to the sentence, storing location and content, then orienting precisely by leveraging the reliable binaural cues for efficiency. SSD groups’ heightened promptness likely reflects the need to initiate searches to navigate complex scenes, not for localization (which remained poor), but to enhance speech intelligibility via head-shadow cues and better ear listening, as evidenced by the preserved outcomes on the intact side (Figure 4). Consequently, shorter “reaction times” may paradoxically indicate increased perceptual ambiguity and listening effort among SSD listeners.

One possible explanation is incomplete plasticity in auditory pathways post-implantation, with cortical reorganization continuing to favor pathways driven by the intact ear (Legris et al., 2018; Kral et al., 2013; Polonenko et al., 2019), which may limit bilateral integration and exacerbate cue mismatches in noise. Although current CI stimulation restores overall auditory activity, it provides spectrotemporally impoverished signals that do not match the fine structure necessary for robust ITD and ILD computation (Grothe et al., 2010; Francart et al., 2018; Bernstein et al., 2021; Zirn et al., 2015). This mismatch may promote down-weighting of the less reliable input rather than stable binaural fusion, consistent with reports of reduced acoustic-ear speech perception in some bimodal listeners after CI use (Svirsky et al., 2020), and asymmetric binaural benefit in acoustic-electric hearing, where degraded electric input can interfere with acoustic processing (Zou et al., 2026). However, direct neurophysiological measures would be needed in future work to test these proposed mechanisms more explicitly. Together, the behavioral findings suggest that structural and perceptual mismatches across ears may limit the precise interaural computations required for meaningful binaural integration.

A limitation of this study is the age difference between the groups. Although age was included in the linear mixed-effects models and was not a significant predictor, the younger NH controls and limited overlap in age distributions prevent us from excluding age-related contributions. Future studies with age-matched controls are needed to better disentangle the effects of hearing status from age.

Behavioral manifestations that may arise from this asymmetry contribute to tangible challenges for SSD-CI users in everyday listening environments. Our results suggest that SSDI-CI listeners prioritize speech intelligibility over localization precision, relying heavily on better-ear listening strategies to manage spatial demands. In dynamic scenarios, SSD-CI users exhibit compensatory behaviors, such as increased head movements and prolonged search times that appear to help resolve spatial ambiguity and improve intelligibility. These adaptive behaviors suggest that listeners actively search to leverage spatial cues when functional binaural hearing remains limited. Although CIs unquestionably restore access to sound on the deaf side and enable coarse lateralization in quiet, the present behavioral data suggest that these benefits do not consistently generalize to spatially complex, dynamic listening conditions in adult SSD recipients. In dynamic real-world scenarios, these individuals may continue to rely on compensatory head-orienting behaviors, suggesting incomplete binaural restoration in single-sided deafness treated with cochlear implants. These findings challenge prevailing narratives of binaural restoration via current CI technology in SSD and highlight the need for novel rehabilitation paradigms that explicitly promote integration of electric input or exploit alternative spatial mechanisms to alleviate behavioral burdens.

## Data Availability

The raw data supporting the conclusions of this article will be made available by the authors, without undue reservation.

## Footnotes

1
https://github.com/sccn/labstreaminglayer
